# Wild Yeast for the Future: Exploring the Use of Wild Strains for Wine and Beer Fermentation

**DOI:** 10.3389/fgene.2020.589350

**Published:** 2020-11-02

**Authors:** Jennifer Molinet, Francisco A. Cubillos

**Affiliations:** ^1^Departamento de Biología, Facultad de Química y Biología, Universidad de Santiago de Chile, Santiago, Chile; ^2^ANID – Millennium Science Initiative Program – Millennium Institute for Integrative Biology (iBIO), Santiago, Chile

**Keywords:** wine, beer, fermentation, *Saccharomyces cerevisiae*, wild strains, hybrids

## Abstract

The continuous usage of single *Saccharomyces cerevisiae* strains as starter cultures in fermentation led to the domestication and propagation of highly specialized strains in fermentation, resulting in the standardization of wines and beers. In this way, hundreds of commercial strains have been developed to satisfy producers’ and consumers’ demands, including beverages with high/low ethanol content, nutrient deprivation tolerance, diverse aromatic profiles, and fast fermentations. However, studies in the last 20 years have demonstrated that the genetic and phenotypic diversity in commercial *S. cerevisiae* strains is low. This lack of diversity limits alternative wines and beers, stressing the need to explore new genetic resources to differentiate each fermentation product. In this sense, wild strains harbor a higher than thought genetic and phenotypic diversity, representing a feasible option to generate new fermentative beverages. Numerous recent studies have identified alleles in wild strains that could favor phenotypes of interest, such as nitrogen consumption, tolerance to cold or high temperatures, and the production of metabolites, such as glycerol and aroma compounds. Here, we review the recent literature on the use of commercial and wild *S. cerevisiae* strains in wine and beer fermentation, providing molecular evidence of the advantages of using wild strains for the generation of improved genetic stocks for the industry according to the product style.

## Introduction

For centuries, humans have mass-produced food and alcoholic beverages using fermentative yeasts, of which wine and beer are the best-known products derived from this process (Steensels and Verstrepen, 2014; Legras et al., 2018; Parapouli et al., 2020). Yeasts are naturally present in fermentation raw materials, such as grape musts (wine fermentation) and cereals (beer fermentation), representing a fraction of the natural microbiota of these carbon-rich environments. However, in most cases, the main microorganisms that could outcompete the others are species belonging to the *Saccharomyces* genus, mainly *Saccharomyces cerevisiae* (Sternes et al., 2017; Stefanini and Cavalieri, 2018; Mandakovic et al., 2020). *S. cerevisiae* is able to dominate the fermentation process, due to its high fermentative capacity and ability to tolerate different stresses, such as high osmotic stress, low pH, high ethanol levels, anerobiosis, and nutrient starvation (Warringer et al., 2011; Brice et al., 2014; García-Ríos et al., 2014; Kitichantaropas et al., 2016; Taymaz-Nikerel et al., 2016; Legras et al., 2018; García-Ríos and Guillamón, 2019). In this sense, most producers use starter cultures in the fermentation process. By the intense re-utilization of yeast, specific *S. cerevisiae* strains were involuntarily selected as starter cultures, guaranteeing a controlled and precise fermentation process and avoiding stuck or sluggish fermentations (Albergaria and Arneborg, 2016). However, the current set of commercial *S. cerevisiae* strains and its derived hybrids is insufficient to provide novel properties to beer and wine, stressing the need for new and improved strains for the industry (Aquilani et al., 2015; Alperstein et al., 2020; Gibson et al., 2020). In recent years, bioprospecting effort has increased the isolation of wild strains from different niches, opening the opportunity to use them (or their genetic variability) in the fermentation process (Duan et al., 2018; Peter et al., 2018; Kang et al., 2019; Pontes et al., 2020).

Here, we review the contemporary literature concerning the use of commercial and wild *S. cerevisiae* strains in the wine and beer industries, highlighting their advantages and disadvantages. Under this scenario, wild strains represent alternative genetic stocks for the industry to overcome current challenges. We review recent progress in the characterization of these strains, new allelic determinants and their performance under wine and beer fermentation conditions, together with their genetic and phenotypic features. In this sense, we defined as “wild strains” those isolated from non-fermentative niches that show no signs of domestication. However, the importance of native strains belonging to domesticated lineages cannot be ignored. In this sense, we highlight the genetic and phenotypic diversity in native and wild strains, providing strategies to improve these genetic stocks for beer and wine fermentations.

## All That Glitters is not Gold: Advantages and Disadvantages of Using Commercial *S. Cerevisiae* Strains

In industrial-scale fermentations, the utilization of starter cultures is preferred over spontaneous inoculations to avoid technical hitches related to slow fermentation rates, end product variability, and yeast contaminants that can spoil the final product (Eliodório et al., 2019; Parapouli et al., 2020). Commercial yeast strains are isolates from fermentation-related environments or are derived from breeding programs, in which they were selected for certain phenotypic traits, such as efficient nitrogen consumption (García-Ríos et al., 2014; Tesnière et al., 2015), fast fermentation rates (Preiss et al., 2018), and pleasant aroma profiles (Eder et al., 2018; Schwarz et al., 2020). These commercial strains usually underwent distinct domestication trajectories, with the exact conditions depending on the traditional practices in the brewery or winery. In this way, traits of industrial interest can differ between distinct lineages, reflecting their specific domestication environment. For example, wine strains have a superior performance in general stress conditions (high sugar and alcohol content) compared to beer strains (Gallone et al., 2016). In contrast, beer strains cover desirable and specific traits for brewing, such as maltotriose utilization, and a lack of production of undesirable off-flavors, such as 4-vinyl guaiacol (Gallone et al., 2016; Krogerus et al., 2017a; Nikulin et al., 2018; Vakirlis et al., 2019). Consequently, industrial beer strains show more notable domestication signatures than wine strains, probably because of the continuous recycling of yeast after each fermentation batch throughout the year (Steensels et al., 2019). In contrast, wine strains only grow and ferment in grape musts during a short period of the year, i.e., 2–3 weeks, and are then forced “back-to-nature.” These differences result in specific genomic features and life cycles in beer and wine strains, as a consequence of selection, domestication, and early hybridization events in ancient lineages.

The genomic characterization of wine and brewing strains demonstrates that their geographical origin and industrial applications have shaped the evolutionary divergence of industrial yeasts (Liti et al., 2009; Borneman et al., 2011; Gallone et al., 2016; Gonçalves et al., 2016; Legras et al., 2018; Peter et al., 2018). In particular, commercial wine strains form a defined phylogenetic cluster, distributed around the world across the Mediterranean, and Mediterranean-like regions (Legras et al., 2007; Liti et al., 2009; Almeida et al., 2015; Peter et al., 2018), and are separated from the *S. cerevisiae* ale strains (Gallone et al., 2016; Gonçalves et al., 2016). Ale strain divergence is complex and is shaped by their clonal life cycle in an industrial niche and by the geographical location of the brewery. They separate into two clades or lineages, Beer 1 and Beer 2. The Beer 1 clade contains Belgium/Germany, Unites States, Britain and kveik strains (Gallone et al., 2016; Gonçalves et al., 2016; Preiss et al., 2018; Pontes et al., 2020). In contrast, the Beer 2 clade lacks a geographical structure, and strains are closely-related to wine strains (Gallone et al., 2016). Interestingly, kveik yeasts form a distinct group related to the Beer 1 clade. However, these strains have a possible mixed ancestry, suggesting a hybrid origin for kveik strains between Beer 1 and an unknown lineage (Preiss et al., 2018). Indeed, further evidence suggests that polyploid brewing strains originated from ancient admixture events between European wine strains and Asian rice wine strains (Fay et al., 2019; Pontes et al., 2020). Altogether, these findings demonstrate that modern commercial strains are the product of unintended historical hybridization events as a result of human selection for specific traits (Fay et al., 2019; Nespolo et al., 2020; Pontes et al., 2020).

Recent genetic studies have demonstrated that commercial wine and beer strains lack genetic and phenotypic diversity, where genetically-similar strains are sold with different commercial names (Fernández-Espinar et al., 2001; Schuller et al., 2004; Dunn et al., 2005; Legras et al., 2005; Borneman et al., 2016; Bindon et al., 2019). In particular, wine strains show a lower nucleotide diversity (*π* = 1 × 10^−3^) compared to beer strains (*π* = 2.8 × 10^−3^) and in the species as a whole (*π* = 3 × 10^−3^; Peter et al., 2018). Although beer strains have a greater genetic diversity than wine strains, they originate from only two ancestors, which is estimated to have occurred between AD 1600 and AD 1700 (Gallone et al., 2016, 2018). The low genetic and phenotypic diversity of these strains may negatively-impact the producer’s perception of the substantial role of the chosen yeast over the final product. Therefore, current genetic stocks limit the diversification of novel beverage properties, increasing the need to search for new alternatives to differentiate each fermentation product (Aquilani et al., 2015). This stresses the desire to explore the genetic and phenotypic diversity in other *S. cerevisiae* lineages to obtain new and novel strains with different and improved fermentative characteristics. In this sense, one source of genetic diversity can be found in non-domesticated *S. cerevisiae* strains collected from the wild.

## Wild *S. Cerevisiae* Strains: Genetic Stocks for Novel Fermentation Products

**Figure 1:**
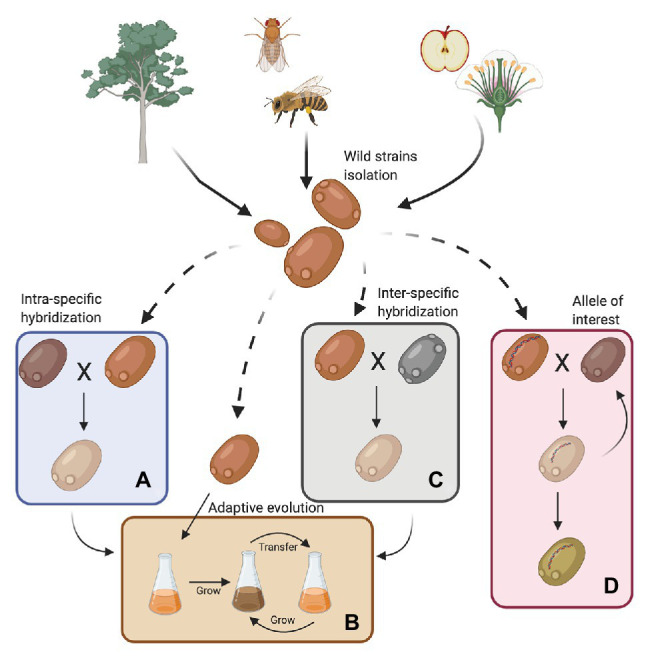
Sources for obtaining wild *Saccharomyces cerevisiae* strains and their use in the fermentation process. Wild strains may be obtained from various environments such as insects, wasps and flies, flowers, trees, and their surroundings. These strains must be phenotypically characterized under fermentative conditions of interest. In this way, strains can then be selected for genetic improvement programs with commercial strains (**A**, intra-specific hybridization), subjected to an adaptive evolution process **(B)** or used in the generation of interspecific hybrids **(C)**. Furthermore, wild alleles may be incorporated into commercial strains by an assisted introgression strategy **(D)**. Finally, strains obtained by hybridization can also be subjected to an adaptive evolution process (Created with BioRender.com).

For years, scientists believed that *S. cerevisiae* was primordially found in fruits or fermentation-related environments. However, bioprospecting efforts have established that it is much more widespread; it has been isolated from wasps, flies, oak trees, and their associated substrates, such as bark, soil, and flowers (Figure 1; Zhang et al., 2010; Alsammar and Delneri, 2020; Pontes et al., 2020). In this way, *S. cerevisiae* follows the nomad model, being able to survive as a generalist at low abundance in a wide range of environments and is not necessarily adapted to a specific niche (Goddard and Greig, 2015). Recent genomic studies, including a large number of wild isolates, demonstrated that the population structure of *S. cerevisiae* is more complex than previously reported (Liti et al., 2006; Legras et al., 2007), with abundant genomic and phenomic diversity (Liti et al., 2009; Warringer et al., 2011; Wang et al., 2012; Bergström et al., 2014; Legras et al., 2018; Peter et al., 2018). These studies have described more than 20 independent lineages in the species, where 13 correspond to wild or non-domesticated lineages (Duan et al., 2018; Peter et al., 2018; Pontes et al., 2020). Overall, wild strains showed lower heterozygosity levels, higher indels rates and sequence diversity, lower gene duplication levels, lower horizontal gene transfer (HGT) events, and lower genome content variation (median 161 shared ORFs vs. 115 ORFs that are not shared; Peter et al., 2018; Kang et al., 2019). Furthermore, wild strains are generally diploid, and their diversity is dependent on SNPs rather than on genomic rearrangements (Peter et al., 2018).

Wild *S. cerevisiae* are genetically and phenotypically separated from industrial *S. cerevisiae* strains, mostly due to the result of human selection (Gallone et al., 2016; Duan et al., 2018; Peter et al., 2018; Pontes et al., 2020). In general, commercial strains produce relatively low levels of extracellular aromatic compounds (such as benzoate, 4-aminobenzoate, and nicotinate) and have low sporulation levels, but exhibit high ethanol and low-pH tolerance (Duan et al., 2018; Kang et al., 2019). In contrast, wild strains have relatively high extracellular levels of different secondary metabolites (such as aspropyl acetate, isoamyl acetate, and ethyl acetate), high fatty acid biosynthesis, and higher energy flux toward the TCA cycle (Kang et al., 2019). These features directly translate into greater production of fatty acids, which are associated with a more robust response to different stresses, such as high salt and ethanol concentrations, high temperatures, and oxidative stress (Qiu et al., 2019). Therefore, wild strains tend to exhibit higher ranges of resistance to multiple conditions, and accordingly, offer significant potential for utilization in a wide range of industrial applications (Kang et al., 2019). Furthermore, industrial strains display selfish behavior with a rapid glucose consumption rate, a large cell size, and nutrient storage inside the cell. In contrast, wild strains display a cooperative behavior and secrete secondary metabolites to poison or cross-feed competitors (Spor et al., 2009). This behavior may be advantageous during fermentation. For example, wild isolates from flowers and sugar-rich sources may produce a different set of volatile compounds, expanding the repertoire of aromas and flavors in alcoholic fermentation (Pontes et al., 2020). In this sense, wine fermentations using indigenous wild strains obtained from oak trees produce earthy and sulfurous organoleptic features, and at the same time, high levels of citrus and floral attributes (Hyma et al., 2011). However, native strains isolated from vineyards, grape, and soil display excellent fermentation properties, but wild isolates from oak barks exhibit stuck fermentation profiles (Camarasa et al., 2011). Therefore, the direct use of wild strains could be limited, and strategies for their improvement are needed.

Although wild isolates may not be useful on their own for fermentative applications, they harbor alleles that could be of interest for breeding programs (Figure 1 and Table 1). For example, low levels of yeast assimilable nitrogen (below 140 mg/L) in grape must are the primary source of sluggish or stuck fermentations (Gobert et al., 2019; Kessi-Pérez et al., 2020a). In this context, recent studies have demonstrated that alleles from a wild *S. cerevisiae* strain might play a decisive role in the adaptation to low nitrogen concentrations (Molinet et al., 2019; Kessi-Pérez et al., 2020a,b). *SAP185*, *TOR2*, *SCH9*, and *NPR1* alleles derived from an oak strain increased amino acid consumption (aspartic acid, histidine, glutamine, and threonine) compared to wine alleles under wine fermentation conditions (Molinet et al., 2019). Another phenotype of interest related to nitrogen consumption is volatile compound production (Swiegers and Pretorius, 2007). In particular, volatile thiols contribute to the typicity of Sauvignon Blanc wines. In this regard, a bulked segregant analysis identified the *IRC7* allele from a clinical isolate as responsible for a higher production of 4-mercapto-4-methylpentan-2-one (4MMP), which contributes boxwood and black currant aromas to wines (Swiegers et al., 2009). This allele is an introgression from *Saccharomyces paradoxus*, while the majority of *S. cerevisiae* strains (including commercial wine strains) harbor a 38-bp deletion that generates a truncated protein. The overexpression of the beneficial allele in a commercial wine strain (Zymaflore F15) increases 4MMP production during Sauvignon Blanc fermentation from below detectable levels (<10 ng/L) to 1,000 ng/L (Roncoroni et al., 2011). Overall, these studies highlight that there is an emerging opportunity to broaden the genetic and phenotypic variability from natural populations, which are still poorly investigated. Hence, non-domesticated wild isolates harbor a valuable source of genetic diversity, which is useful for domestication and breeding programs to generate novel strains or hybrids for the wine and beer fermentation industries.

**Table 1:** Examples of alleles from wild or non-domesticated strains identified in QTL mapping studies with a beneficial impact on different phenotypes of interest.

Phenotype	Gene(s) identified	Strain(s)	Reference(s)
Amino acid consumption	*SAP185, TOR2, SCH9, and NPR1*	YPS128 (oak isolate)	Molinet et al., 2019
Acetic acid production	*ALD6*	YPS128 (oak isolate)	Salinas et al., 2012
Sugar consumption	*MBR1, HAP4*	YPS128 (oak isolate)	Salinas et al., 2012
Thiol production (4-mercapto-4-methylpentan-2-one)	*IRC7*	YJM450 (clinical isolate)	Roncoroni et al., 2011
Growth under nitrogen limited conditions	*ECM38, DAL80*	YPS128 (oak isolate)	Kessi-Pérez et al., 2020b
Heat sensitivity	Subtelomeric region Chr XIII-R	YPS128 (oak isolate)	Cubillos et al., 2011
Heat stress	*IRA1, IRA2*	YPS128 (oak isolate)	Parts et al., 2011; Cubillos et al., 2013
Sporulation efficiency	*RME1, IME1, RSF1*	YPS606 (oak isolate)	Gerke et al., 2006, 2009
Mycotoxin (mycophenolic acid) susceptibility	*IMD2*	YPS128 (oak isolate)	Quispe et al., 2017
Glycerol production	*GPD1, SSK1*	YPS128 (oak isolate), CBS6412 (unknown)	Hubmann et al., 2013; Tapia et al., 2018
Freeze-thaw stress	*AQY1, AQY2*	YPS163 (oak isolate)	Will et al., 2010
Growth-limiting glucose concentration	*HXT7*	BC248 (oak isolate)	Ziv et al., 2017
Near-freezing temperature tolerance	*NAT1*	ZX_11_(6; Chinese isolate)	Feng et al., 2018
Chronological lifespan	*HPF1, FLO11*	YPS128 (oak isolate)	Barré et al., 2020

## Exploiting Genetic Variants From Wild *S. Cerevisiae* Isolates to Generate Novel Hybrid Strains for Beer and Wine

Wild strains with genetic variants of interest could be used in breeding-related programs to generate genetically-improved strains (Figure 1; Steensels et al., 2014b). Intra-specific hybridization (two strains from the same species) has been successfully applied in *S. cerevisiae*, improving different traits for wine fermentation, such as low sulfur compound levels (Agarbati et al., 2020), novel aroma profiles (Marullo et al., 2006; Steensels et al., 2014a), improved stress tolerance (Bonciani et al., 2016), wider temperature tolerance (Marullo et al., 2009), fermentation of nitrogen-deficient musts (Kessi-Pérez et al., 2020c), and lower ethanol production (García et al., 2012). Similarly, breeding strategies have generated hybrids exhibiting high beer fermentation performance and producing different aroma profiles, likely due to transcriptional cross-talk (Gallone et al., 2016).

Interspecific hybrids between *S. cerevisiae* and *S*. non-*cerevisiae* strains are easily isolated from fermentative environments (González et al., 2006, 2008; Querol and Bond, 2009; Peris et al., 2012). Genetic studies highlight that the *S*. non-*cerevisiae* parental portion has a wild origin (Gallone et al., 2019; Langdon et al., 2019). In this way, it is possible to use wild *Saccharomyces* strains for the generation of new commercial hybrid stocks, and thus expand the genetic and phenotypic diversity. Interspecific *Saccharomyces* hybrids are possible to find due to a weak pre-zygotic barrier between species (Alsammar and Delneri, 2020). In contrast, post-zygotic barriers impede successful meiosis, and spore viabilities in hybrids are typically below 10% (Liti et al., 2006; Hittinger, 2013), limiting the generation of recombinant hybrids between species. Interspecific *Saccharomyces* hybrids inhabit different fermentation environments, exhibiting interesting and complex genomic compositions (Alsammar and Delneri, 2020). In this sense, recent reports show the existence of four types of hybrids determined by their geographical origin and industrial practices: *S. pastorianus* associated with beer; *S. cerevisiae* × *Saccharomyces kudriavzevii* associated with beer and wine; *S. eubayanus* × *Saccharomyces uvarum* associated with different environments; and complex hybrids with three or four parental species: *S. cerevisiae* × *S. kudriavzevii* × *S. eubayanus* × *S. uvarum*, *S. cerevisiae* × *S. eubayanus* × *S. uvarum* and *S. cerevisiae* × *S. kudriavzevii* × *S. eubayanus* (Gallone et al., 2019; Langdon et al., 2019). In all cases, the *S. cerevisiae* parental strains belong to three domesticated lineages (Wine, Beer 1, and Beer 2), providing traits and fermentative advantages when used in industrial settings. These reports highlight the importance and relevance of interspecific hybridization in the diversification and adaptation of yeast to industrial niches.

Interspecific hybridization is an evolutionary strategy that allows swift adaptation to new niches (Gallone et al., 2019). In this context, the construction in the laboratory of novel hybrids using wild strains showing improved fermentation performance compared to their parental species, is an attractive strategy for future breeding projects (Figure 1). For example, *de novo* hybridization of *S. cerevisiae* × *S. eubayanus* strains combined the sugar utilization properties of *S. cerevisiae* and the cryotolerance of *S. eubayanus* (Hebly et al., 2015; Krogerus et al., 2017b). In this sense, the utilization of all *Saccharomyces* species for *de novo* hybridization with *S. cerevisiae* has the potential to increase the genetic diversity of yeasts for the wine and beer industries (Brickwedde et al., 2018; Nikulin et al., 2018). However, one of the limitations observed in studies that generate *de novo* hybrids is the use of a restricted number of parental strains. The utilization of a handful of isolates per species is not necessarily representative of the phenotypic spectrum of the progeny generated by hybridizing individuals from two (or more) different species. In this way, taking advantage of the full genetic diversity already described in the *Saccharomyces* genus would be a promising step toward developing new yeast hybrids. Therefore, wild isolates are strong candidates for innovations in the industry (Cubillos, 2016; Cubillos et al., 2019). However, it is important to note that such polyploid hybrids tend to be genetically unstable, and may undergo extensive changes after hybridization (Gallone et al., 2018), which opens the opportunity to improve and adapt them to conditions found in fermentative processes. Consequently, hybrids have to reach genomic stability under conditions generally encountered during alcoholic fermentation (Krogerus et al., 2018). Experimental evolution approaches are the right strategies to follow in order to reconstruct evolutionary trajectories of novel hybrid strains subjected to fermentation environments (Krogerus et al., 2018). Different studies utilizing experimental evolution provide evidence of the genetic changes taking place during adaptation to fermentation, including partial loss of one of the parental subgenomes, loss of heterozygosity, selection of superior alleles, and the formation of fusion genes following translocations (Piotrowski et al., 2012; Dunn et al., 2013; Hope et al., 2017; Peris et al., 2017; Smukowski Heil et al., 2017).

## Conclusion

The standardization of wines and beers as a result of the utilization of a genetically reduced set of commercial strains has brought with it the need for new and novel products that can be highlighted and differentiated. In this sense, wild strains and genetically diverse interspecific hybrids are an attractive alternative for the industry. However, challenges persist in adapting and improving wild strains to fermentative environments. Such challenges could be overcome through genetic improvement programs together with adaptive evolution strategies. The generation of new strains and intra‐ and inter-species hybrids could open up new avenues in order to obtain unique strains for the wine and beer industries.

## Author Contributions

All authors listed have made a substantial, direct and intellectual contribution to the work, and approved it for publication.

### Conflict of Interest

The authors declare that the research was conducted in the absence of any commercial or financial relationships that could be construed as a potential conflict of interest.
